# Spatio-spectral beam control in multimode diode-pumped Raman fibre lasers via intracavity filtering and Kerr cleaning

**DOI:** 10.1038/s41598-021-01491-0

**Published:** 2021-11-09

**Authors:** Sergey A. Babin, Alexey G. Kuznetsov, Oleg S. Sidelnikov, Alexey A. Wolf, Ilya N. Nemov, Sergey I. Kablukov, Evgeniy V. Podivilov, Mikhail P. Fedoruk, Stefan Wabnitz

**Affiliations:** 1 Institute of Automation and Electrometry SB RAS, 1 Ac. Koptyug ave., Novosibirsk, 630090 Russia; 2 Novosibirsk State University, 2 Pirogova Str., Novosibirsk, 630090 Russia; 3 DIET, Sapienza University of Rome, Via Eudossiana 18, 00184 Rome, Italy

**Keywords:** Medical research, Optics and photonics

## Abstract

Multimode fibres provide a promising platform for boosting the capacity of fibre links and the output power of fibre lasers. The complex spatiotemporal dynamics of multimode beams may be controlled in spatial and temporal domains via the interplay of nonlinear, dispersive and dissipative effects. Raman nonlinearity induces beam cleanup in long graded-index fibres within a laser cavity, even for CW Stokes beams pumped by highly-multimode laser diodes (LDs). This leads to a breakthrough approach for wavelength-agile high-power lasers. However, current understanding of Raman beam cleanup is restricted to a small-signal gain regime, being not applicable to describing realistic laser operation. We solved this challenge by experimentally and theoretically studying pump-to-Stokes beam conversion in a graded-index fibre cavity. We show that random mode coupling, intracavity filtering and Kerr self-cleaning all play a decisive role for the spatio-spectral control of CW Stokes beams. Whereas the depleted LD pump radiation remains insensitive to them.

## Introduction

It is well-known that stimulated Raman scattering on molecular vibrations not only shifts the wavelength of laser radiation, but may also improve laser beam quality. Initially driven by applications such as laser fusion^1^, Raman beam cleanup (RBC) has been extensively studied since its first demonstration in 1979^2^, as it provided an effective solution for improving quality and brightness of high-power Nd:glass or excimer laser beams via Raman conversion in gaseous^2–6^ or solid-state^7–9^ media.

This idea was further applied to fibre lasers, which became superior to other types of lasers in the 2000s due to their ultimate efficiency, beam quality and thermally mitigating architecture^10^. In contrast to rare-earth-doped fibre lasers operating in a limited wavelength range near 1 (Yb), 1.5 (Er) or 2 (Tm,Ho) µm, respectively, Raman fibre lasers (RFL) may operate almost at any wavelength in the transmission window of passive silica fibres, and benefit from RBC whenever multimode fibres are used^11–13^. A small-signal gain analysis, based on pump and Stokes’ modes overlap integrals^13^, has shown that the RBC effect is present in graded-index fibres (GIFs), since lower-order Stokes modes experience higher Raman gain under random pump launching conditions. Whereas in step-index fibres (SIFs) all transverse modes have nearly the same Raman gain. This theoretical understanding remains only qualitative, and it is not applicable to describing realistic multimode fibre Raman lasers, operating in the strong signal generation regime.

Interest in multimode RFLs was greatly increased in the 2010s, thanks to new perspectives of using multimode fibres for optical communications^14^ and high-power lasers^15^. Although multimode fibres exhibit a rich and complex mix of spatial and temporal nonlinear phenomena^16,17^, nonlinear spatial beam cleaning effects in GIFs enable the stabilization and control of transverse laser beams, which is extremely important for beam delivery applications, especially in pulsed regimes^18,19^. Besides, it becomes possible to directly pump multimode RFLs by high-power continuous-wave (CW) multimode LDs^20–23^. Remarkably, high-beam-quality output Stokes beams may be generated with GIFs, thanks to RBC and possibly additional nonlinear effects, leading to significant brightness enhancement. The output beam quality approaches the diffraction limit^24^, whereas the output power can be at kilowatt levels in all-fibre amplifier and laser configurations^25,26^. One of the key elements of multimode RFLs is the fibre Bragg grating (FBG) with spatially tailored structure, which became possible thanks to the femtosecond (fs) inscription technology^27^. Therefore, multimode RFLs become a very attractive class of CW high-power fibre lasers, potentially free of drawbacks inherent to rare-earth-doped fibre lasers such as: limited wavelength range, photo-darkening, and transverse-mode instability^10,15^. At the same time, there is still a lack of fundamental knowledge about mechanisms of brightness enhancement in the Raman conversion of highly-multimode broadband CW LD radiation.

Here we experimentally and theoretically study the interplay of different physical processes in the intracavity interaction of LD pump and Stokes beams in multimode GIFs. We identify the role of both Raman and Kerr nonlinearity, inhomogeneous pump depletion, random mode coupling and intra-cavity filtering for spatio-spectral beam control. This permits to unveil the conditions for efficient conversion of highly-multimode LD pump beams into a nearly-singlemode Stokes beam.

## Results

**Figure 1:**
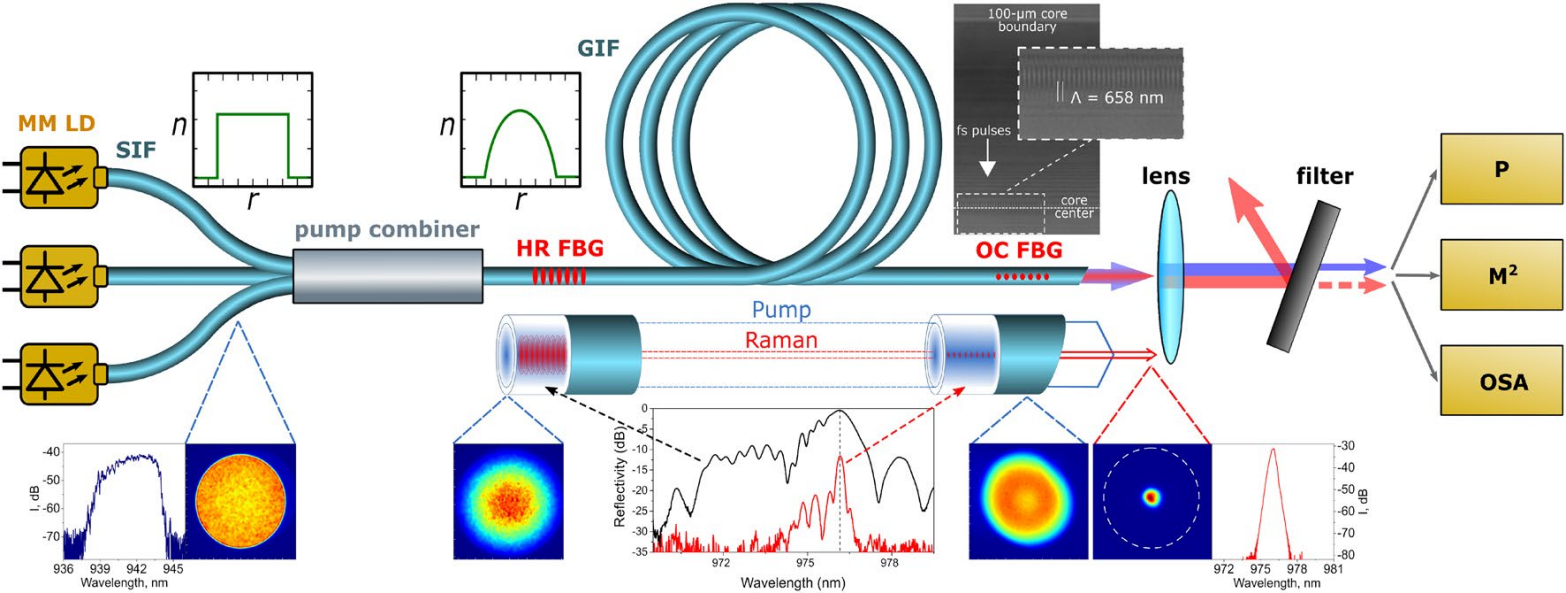
Multimode fibre Raman laser scheme: three CW multimode laser diodes (MM LD) pigtailed by SIF with 100-µm core are combined by a pump combiner and pump a 1-km GIF with 100-µm core, where a resonator is formed by an UV-inscribed highly-reflective (R ~ 90%) fibre Bragg grating (HR FBG) and a fs-inscribed output coupler (R ~ 4%) (OC FBG), playing the role of a 10-dB filter for the fundamental mode, due to localization near the GIF axis (the OC FBG microscopic image is shown at the top, while OC and HR FBGs reflection spectra are shown at the bottom). The laser output is collimated by a lens, and characterized by a power meter (P), beam profiler (M^2^) and optical spectrum analyzer (OSA), with the use of corresponding interference filters for selection of either the pump (940 nm) or the Stokes (976 nm) beam. Beam spectrum and cross-sections for the multimode LD pump radiation measured before and after pump combiner in SIF and GIF, respectively, and after OC FBG, are shown together with the generated Stokes beam shape and spectrum at the output.

The design of the multimode LD-pumped GIF Raman laser is shown in Fig. 1 together with the characterization scheme (see “Methods” for details). Along the bottom border of the drawing we show how the spatial (beam shape) and spectral (optical spectrum) characteristics evolve in the multimode fiber. The pump beam from an individual LD almost homogeneously fills the core of the LD pigtail made of SIF so that the transverse intensity profile of the pump beam becomes to be similar to the SIF index profile (the measured beam quality parameter here is M^2^ ≈ 26). The colored intensity diagram exhibits only weak signs of speckles that may be reasoned by the large bandwidth (~ 5 nm) of LD radiation. The pump beam quality slightly degrades (M^2^ ≈ 34) after the mixing of three beams coming from individual LDs in the spliced fibre combiner and the propagation of the combined beam in ~ 2 m output port of the combiner made of GIF. At the same time, the transverse intensity profile of the pump beam approaches to a parabolic shape that mimics the index profile of GIF core. Note that the parabolic beam shape corresponds to an equipartition of pump energy among all of the transverse modes, which is reached due to a strong random mode coupling in spooled fiber (see Supplementary Note 2). The pump beam propagates in GIF without sufficient distortions and its shape remains nearly parabolic at the fiber output. However, above the Raman threshold the transmitted pump beam gets depleted owing to the pump-to-Stokes beam conversion. The generated Stokes beam is much narrower than the pump beam that leads to a spatial hole burning in the central part of the pump beam. The Stokes beam remains narrow both in the spatial and spectral domains up to the maximum power value.

**Figure 2:**
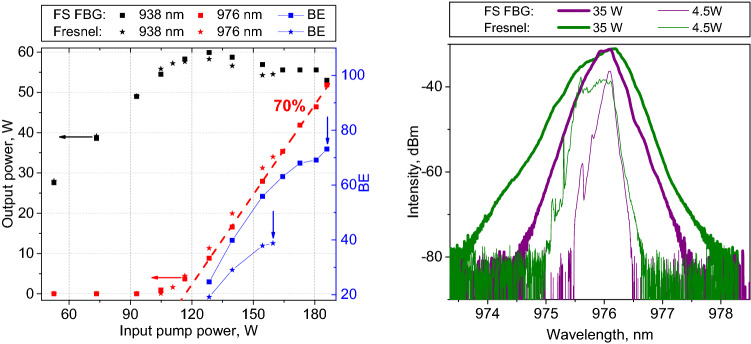
Comparison of output characteristics in two RFL configurations with OC based on fs-inscribed FBG and Fresnel reflection, respectively: (**a**) Pump (940 nm) and Stokes (976 nm) power at the output and pump-to-Stokes brightness enhancement (BE) factor as a function of input pump power up to the 2nd Stokes threshold at which 2nd Stokes power reaches ~ 50 mW (vertical arrow); (**b**) Stokes spectra at power of 4.5 W and 35 W in the two configurations.

In order to identify the role of FBG filtering on the Stokes generation process, we replace the OC based on fs-FBG (4% narrowband reflection for the fundamental mode, and ≤ 0.4% for higher orders, see bottom inset in Fig. 1) by the Fresnel reflection from a normally cleaved fibre end without fs-FBG (~ 4% broadband reflection for all modes), with identical other components. For both configurations, we measured output power, spectra, beam quality parameter M^2^ and beam profiles at the output end facet of the GIF (see “Methods” and Supplementary Note 1 for details). From these data, we calculated values of the pump-to-Stokes brightness enhancement factor BE = [P/(M^2^λ)^2^]_Stokes_/[P/(M^2^λ)^2^]_Pump._ BE is ~ 2 times higher in the case of a fs-FBG, reaching BE = 73 at maximum power of 52 W (Fig. 2a), due to improved beam quality (M^2^ = 2.02 at the maximum power) provided together with narrow bandwidth (< 0.4 nm) by the spatio-spectral filtering property of the fs-FBG (Fig. 2b). Note that the BE value obtained here is higher than in our previous experiments with fs-FBG in a similar RFL scheme^28^, where BE = 68 was reported.

**Figure 3:**
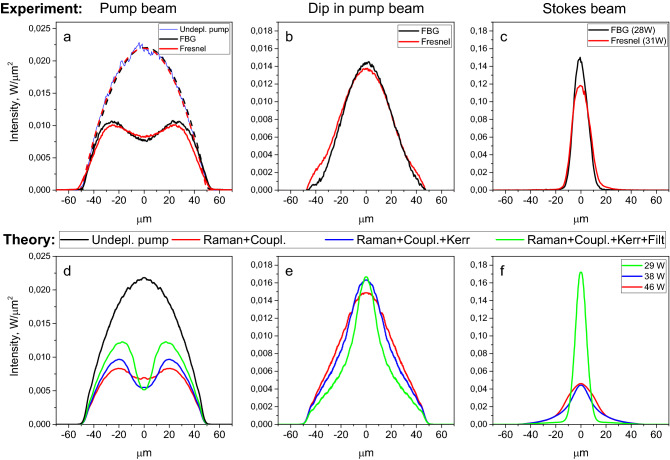
Comparison of experimental beam profiles in two RFL configurations with OC based on fs-inscribed FBG and Fresnel reflection: (**a**) experimental profiles for output pump beam and undepleted pump beam (input beam with an account for fibre attenuation) and its parabolic fit in the two cases (dashed lines); (**b**) dip in the pump beam determined as the difference between the udepleted and output pump beam profiles; (**c**) experimental profile for the output Stokes beam. Corresponding theoretical profiles: (**d**) pump beam; (**e**) dip in the pump beam; (**f**) Stokes beam, which are calculated with an account for different processes (random mode coupling, Kerr effect, FBG filter) in addition to Raman effect—different combinations are marked by different colors. Both the experimental and the theoretical data are obtained for input pump power *P*_*in*_ ≈ 154 W.

We compared experimental beam intensity profiles for two configurations, at the input pump power *P*_*in*_ ≈ 154 W. The output pump beam profile (Fig. 3a) and the depleted dip (Fig. 3b)—or difference between the output and input pump profiles, when taking into account fibre attenuation—remain nearly unchanged. At the same time, the output Stokes beam profile (Fig. 3c) is narrower in case of fs-FBG, in accordance with the better M^2^ value.

The same profiles were simulated on the basis of the coupled-mode model^29^, taking into account the physical processes specific to a LD-pumped GIF RFL (see “Methods”). For identifying the role of individual effects, we carried out simulations by adding them sequentially, with the input pump beam intensity profile kept parabolic (Fig. 3d), in accordance with experimental results. Pure Raman conversion gives a speckled structure of pump and Stokes beams (see Supplementary Note 2), while in the experiment they remain smooth. Adding random mode coupling [red curves in Fig. 3d–f)] gives smooth beam profiles as in experiments, but the simulated Stokes beam remains broad, with only 7% fundamental mode (FM) content. The inclusion of the Kerr effect (blue curves) significantly compresses the Stokes beam width, a signature of beam self-cleaning^18,29^: the FM content increases by ~ 4 times from 7 to 31%, in correspondence with the beam width reduction, resulting from a substantially lower high-order modes content when evaluated by radial integration. At last, adding a spatial 10-dB filter selecting the fundamental mode permits to further improve the Stokes beam quality, resulting in higher intensity at similar powers (with a FM content up to ~ 70%), in agreement with experiments. Note that, in the experiments, the difference between filtered and unfiltered Stokes beams is not as large as in simulations, which can be attributed to the additional filtering effect by HR FBG (of ~ 3 dB, see Fig. 1), which was not taken into account in our simplified model. This is justified by the fact that the main essence of our numerical studies was the determination of the respective influence, on the spatial profiles of pump and Stokes beams, of nonlinear (as opposed to linear) spatio-temporal shaping effects such as Raman and Kerr beam cleanup. In contrast to the Stokes beam (Fig. 3f), which is very sensitive to all factors, the dip in the pump (which is spatially much broader than the Stokes beam (Fig. 3e) remains weakly affected.

## Discussion

**Figure 4:**
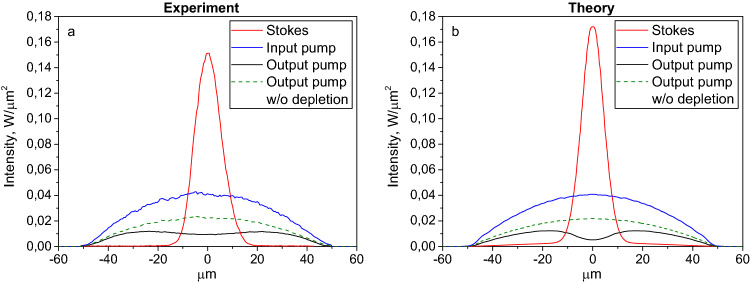
Comparison of intensity profiles for the pump and Stokes beams: experiment (**a**) and theory (**b**) at ~ 154 W input pump and ~ 30 W output Stokes power. To calculate the output pump without depletion, we multiply the input pump profile by the corresponding linear attenuation factor of the fibre.

By comparing experimental intensity profiles with theory accounting for all key physical processes (Fig. 4), we can see a good correspondence. This demonstrates that the broad depleted dip in the pump is converted into a narrow and high-intensity, nearly-singlemode Stokes beam that results in brightness enhancement. In spite of the presence of strong random mode coupling, the Stokes beam is not washed out, thanks to the combined action of Kerr self-cleaning and FBG filtering. This is in contrast with the highly-multimode LD pump beam, whose transverse profile is mainly driven by random mode coupling, leading to parabolic shape and to nearly homogeneous depletion via Raman conversion in the long GIF.

In conclusion, we experimentally and theoretically clarified the mechanisms of spatio-spectral beam control in CW multimode LD-pumped Raman lasers based on GIF with a FBG cavity, operating far from the small-signal regime. The highly-multimode pump beam experiences strong randomization in multimode fibres due to mode coupling: it acquires a nearly rectangular profile in SIFs, and parabolic profile in GIFs, which correspond to equipartition of modes with corresponding index profile. A parabolic pump profile with maximum at the axis leads to predominant amplification of low-order Stokes modes near the Raman threshold. Well above the threshold, the pump beam becomes depleted, but mode coupling washes-out the dip in its center. At the same time, the nearly-singlemode Stokes beam remains almost undisturbed, thanks to Kerr self-cleaning and fs-FBG-induced spatio-spectral filtering, which compensate for randomization. It should be noted that in FBG-free kW-level Raman amplifiers^25^, the Kerr effect alone should play a significant role in the observed beam cleanup. Remarkably, the CW Stokes power in RFLs is about two orders of magnitude lower than that in amplifiers^25^, as well as the typical power level which is required for Kerr-cleaning of sub-nanosecond pulses in ~ 10 m of GIF^18,29^. This means that in a km-long fbre cavity the Kerr self-cleaning effect may already enter into play at tens of Watts power levels.

We have shown that a GIF with in-fibre FBGs is a very simple and robust converter of highly-multimode LD pump light into nearly-singlemode Stokes radiation, with ~ 70% slope efficiency, and brightness enhancement factor approaching to 80. This opens the way to high-power fibre lasers at wavelengths ≤ 980 nm, with frequency doubling into the blue range. The significant power scaling capabilities^25,26^ and inherent tunability of LD-pumped RFL may lead to widespread use in environmental, cultural heritage, biomedical and material processing applications. Nonlinear optical control of spatio-spectral beam parameters will provide a new platform of wavelength-agile high-power LD-pumped light sources.

## Methods

### Experimental set-up

In the all-fibre Raman laser, we used a standard, commercially available GIF of ~ 1 km length, 100 µm core diameter and 0.29 NA, see Fig. 1. The fibre was intentionally wound and bent in a fibre bundle to excite a large number of guided modes (∼ 2000). Three pump laser diodes (λ_P_ ~ 940 nm) were connected to a 3 × 1 fibre pump combiner with SIF 105-µm core input ports and a 100-µm core GIF output port. The combined pump radiation with total power up to ~ 200 W was coupled into the GIF, in which a laser cavity is formed by a UV-inscribed highly-reflective (R ~ 90%) FBG at the input end, with output coupler (OC) based on fs-inscribed FBG (R ~ 4%) with angled cleaved fibre end. Femtosecond point-by-point writing technology offering new opportunities for multimode fibres^27^ was used to create a 2nd-order FBG in the near-axis area (~ 5 µm) of the graded-index core, see microscope image in Fig. 1. This FBG acts as spatial filter, since predominant reflection (R ~ 4%) occurs for the fundamental transverse mode with 10 dB lower reflection coefficient (R ≤ 0.4%) for higher order modes, see reflection spectra in the bottom inset of Fig. 1. At the same time, the UV-inscribed HR FBG reflects several low-order groups of degenerate modes with ~ 3 dB lower reflection coefficient for higher-order modes. As the HR FBG is of the 1st order, it is not seen in microscope, but indirect measurements confirm that its transverse dimension is much larger than that for the fs-inscribed OC FBG.

We characterized pump and Stokes beams (selected by appropriate interference filters) by measuring their power, spectra, beam quality parameter M^2^ and transverse intensity profiles in two cavity configurations: (1) HR FBG + fs-inscribed OC FBG and (2) HR FBG + Fresnel reflection from normally cleaved fibre end (with R ~ 4% for all transverse modes). The transmitted pump power initially grows linearly with increasing input power (*P*_*in*_), with a slope defined by fibre attenuation (~ 2.7 dB), see Fig. 2. Above Raman threshold (*P*_*in*_ ~ 105 W), the transmitted pump power remains nearly constant while the Stokes power at 976 nm grows nearly linearly with input pump power, until it reaches 52 W at the 2nd Stokes threshold (*P*_*in*_ = 186 W) for a fs-FBG OC. A similar behavior is observed for a Fresnel OC: in this case, a lower 2nd Stokes threshold is obtained, which limits to 34 W the maximum Stokes power at 976 nm. Output spectra (Fig. 2b) obtained with a Fresnel OC are > 2 times broader (at their − 3 dB level), for both low and high powers. In addition, these spectra exhibit the presence of additional peaks separated by ~ 0.5 nm, associated with the presence of higher-order mode groups. Therefore, the measured beam quality for a Fresnel OC is worse than that for fs-FBG: at ~ 10 W Stokes power, one obtains M^2^ ≈ 2.2 and 1.7, respectively (see Supplementary Note 1 for details). M^2^ values slightly increase (by ~ 10%) as the power grows to the maximum. The values of pump-to-Stokes brightness enhancement factor BE = [P/(M^2^λ)^2^]_Stokes_/[P/(M^2^λ)^2^]_Pump_ were calculated with the measured P and M^2^ values (see Fig. 2a). Details on the beam profiles measurement procedures together with the obtained results are given in Supplementary Note 1.

### Numerical simulations

We numerically solved a system of coupled mode propagation equations for simulating the spatial evolution of the modal amplitudes of the pump wave and the Stokes component in a GIF. These equations were derived from the coupled system of multidimensional nonlinear Schrödinger equations for the field envelopes of the pump and Stokes beams, by projecting the fields on the basis of Laguerre modes, and neglecting terms which are rapidly oscillating along the propagation direction. The coupled mode equations take into account the effects of self-phase modulation, cross-phase modulation, stimulated Raman scattering, and linear loss (α_s_ = 2.64 dB/km for the Stokes and α_P_ = 2.72 dB/km for the pump wave, respectively). In addition, we also included terms describing random linear coupling between all spatial modes, owing to fibre imperfections, bends and stresses. We used an integration step of 100 mm. In simulations, we include up to 496 modes with mode number n = 2p +|m|≤ 30.

As the initial conditions for the Stokes wave, we used the decomposition of a Gaussian beam with a radius of 12 μm into spatial modes, and set a random input phase for each mode. Calculations were performed for different realizations of random phases, and results were averaged. In our simulations, multiple passes in the linear laser cavity were considered. All pump modes were initialized with equal intensities and random phases at the input of each pass.

We propagated the transverse modes of the beam in the GIF, under the influence of Raman amplification, random mode coupling and Kerr nonlinearity. The intra-cavity Raman conversion of the pump into the Stokes beam in the cavity was computed either without (4% OC for all modes) or with spatial filtering (OC with 4% for FM, 0.4% for higher order modes) before the next pass, as provided by a fs-FBG at each round trip in the cavity (for details see Supplementary Note 2).

## Supplementary Information

Supplementary Information.
