# Supplementary material for: Spatio-spectral beam control in multimode diode-pumped Raman fibre lasers via intracavity filtering and Kerr cleaning
Source: Sci Rep. 2021 Nov 9;11:21994. doi: 10.1038/s41598-021-01491-0 (PMC8578583; doi:10.1038/s41598-021-01491-0)
Supplement: Supplementary file 1 — Supplementary Information. [file 41598_2021_1491_MOESM1_ESM.pdf]

Supplementary Information to:

Spatio-spectral beam control in multimode diode-pumped Raman fibre lasers via intracavity filtering and Kerr cleaning

Sergey A. Babin[1,2*], Alexey G. Kuznetsov[1], Oleg S. Sidelnikov[2], Alexey A. Wolf[1,2], Ilya N. Nemov[1], Sergey I. Kablukov[1], Evgeniy V. Podivilov[1,2], Mikhail P. Fedoruk[2], Stefan Wabnitz[2,3]

[1]*Institute of Automation and Electrometry SB RA, 1 Ac. Koptyug ave., Novosibirsk, 630090, Russia*

[2]*Novosibirsk State University, 2 Pirogova str., Novosibirsk, 630090, Russia*

[3]*DIET, Sapienza University of Rome,,Via Eudossiana 18, 00184 Rome, Italy*

**babin@iae.nsk.su*

Supplementary Note 1. Experimental technique for beam profile measurement:

The beam measurement scheme is shown in fig. S1. The output Stokes signal and residual pump were collimated by a lens L_1. To analyze the effect of pump beam depletion and corresponding Stokes beam parameters we used interference filters IF (with transmission either at 976nm or 1019 nm), corresponding waves were registered by a camera of a Thorlabs M^2- measurement system. To measure a detailed beam profile at the output facet of the fibre we should adjust the fibre position relative to the lens L1 focal point so that the sharp fibre end facet image could be captured by the camera. Using an artificial defect on the test fibre end-face [S1] we have found the optimal fibre position z at which a sharp dip was observed, whereas defocusing from this position blurred out spatial details of the beam. We have also tested by placing an adjustable aperture D that any loss of radiation (even at low-intensity edge tails) leads to significant distorsions of the measured beam profile, so we have taken necessary measures to prevent such losses and to measure the whole beam.

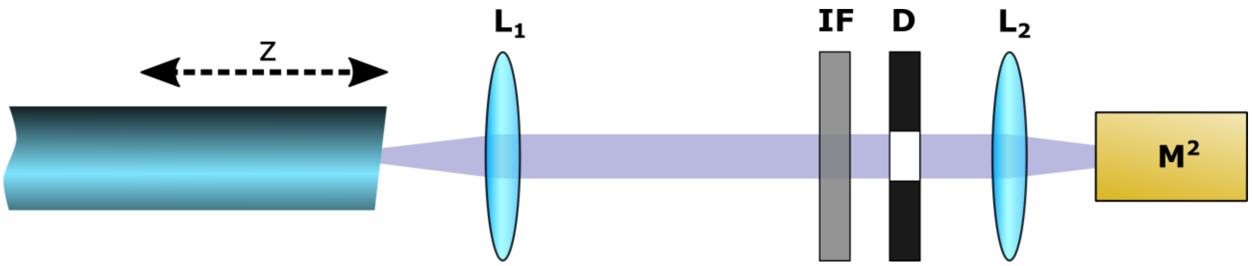

Fig. S1. Beam profiles measurement scheme: the fibre may be moved along the axis z, lenses L_1 and L_2 (internal lens of M^2 meter) transfer an image of the beam from the fibre end facet onto the photomatrix of the M^2 meter, different interference filters are used to select either pump (940 nm) or Stokes (976 nm) beams passed through diaphragm D.

The measured beam profiles together with the beam waist radius as a function of longitudinal position along the focused beam with the fitted M^2 values are shown in Fig.S2. The output pump beam below Raman threshold (Fig.S2a,b) has maximum intensity in the center with near-parabolic profile (see Fig.3 in the main text), and its quality parameter is measured to be $M^2 \approx 34$. Well above threshold, when Stokes power reaches ~10 W, the visible dip is depleted in the pump beam, due to its conversion into the Stokes wave. At the same time, the generated Stokes beam has better characteristics in the case of fs-FBG OC with spatial filtering effect providing $M^2 \approx 1.7$ (Fig.S2c,d,e). Whereas with Fresnel OC the beam quality is worsened to $M^2 \approx 2.2$, and the Stokes beam is visibly broader (Fig.S2f,g,h). Note that in the case of fs-FBG OC the output fibre end is cleaved at an angle of ~10 degrees, in order to eliminate Fresnel reflection. As a result, the measured output beam profiles have slight asymmetry, whereas in case of Fresnel reflection from normally cleaved fibre end all profiles are fully symmetric. Nevertheless, we have justified that this effect does not have an influence on the intensity profiles which we compare for the two configurations (Fig.3 of the main text). We have also checked that the dip shape is correctly extracted from the input and output pump beam profiles. For that purpose, we have compared the power in the dip (depleted pump) with the power in the Stokes beam, both obtained as integral

over the corresponding profile. The obtained data are compared in Fig.S3 for two configurations (fs-FBG OC and Fresnel OC). One can see that the depleted pump and Stokes power values are in good correspondence in the whole power range for both configurations. Non-zero pump depletion near the Raman threshold (Pin~110 W) is explained by the influence of the Stokes amplified spontaneous emission.

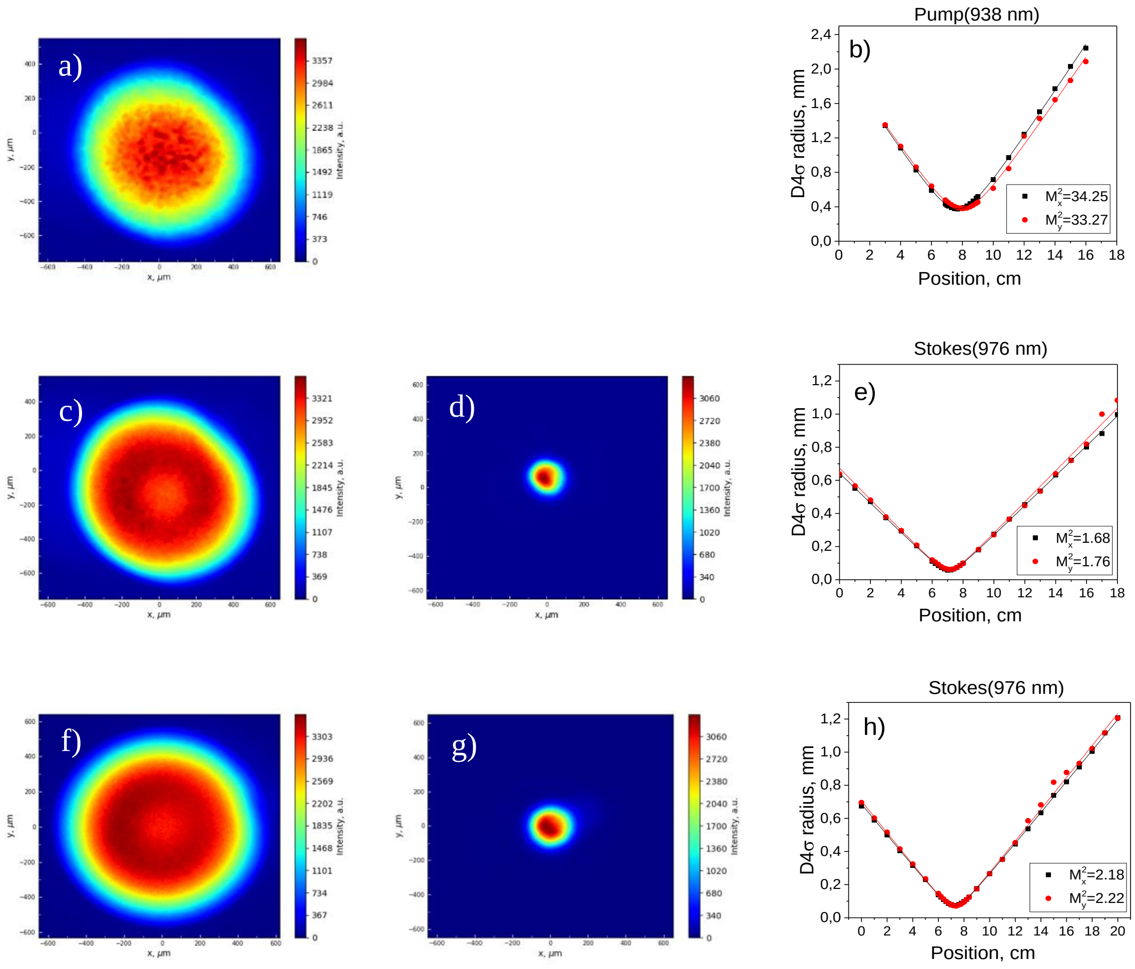

Fig.S2. Output beam profiles and M^2 measurements: pump power below Raman thresholds, P_{in}=20 W (a,b); output pump and Stokes beam profiles and M^2 measurements above Raman threshold at ~10W Stokes power in configuration with fs-FBG OC (c,d,e) and without fs FBG using Fresnel reflection from the output fibre end facet as OC, (f,g,h) at the input pump power P_{in}=130 W.

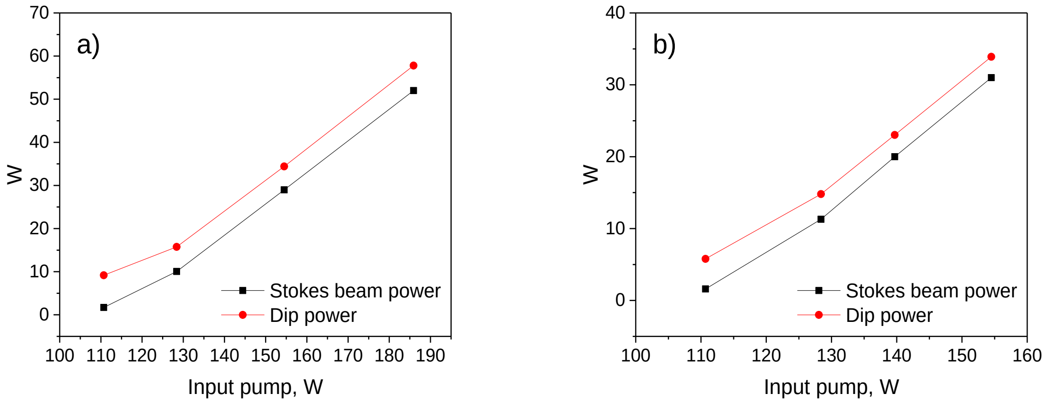

Fig.S3 (b) Comparison of the integrals for dip in the pump profile and for the Stokes beam profile in two configurations with different output couplers: fs-FBG (a), Fresnel reflection (b).

Supplementary Note 2. Theoretical model and numerical solution:

We consider a graded-index multi-mode fibre with the following refractive index profile:

$$n(r) = \begin{cases} n_{co}\sqrt{1-2\Delta(r/R)^2}, & r < R \\ n_{cl}, & r \geq R \end{cases},$$

The propagation of the pump wave and the Stokes component in graded-index (GRIN) multimode fibre (MMF) can be described by the coupled system of multidimensional nonlinear Schrödinger equations:

$$-2ik_S\frac{dA^S}{dz} + \frac{d^2A^S}{dr^2} - 2k_S^2\Delta\left(\frac{r}{R}\right)^2 A^S = -2k_S^2\frac{n_2}{n_0}(|A^S|^2 + (2+if)|A^P|^2)A^S,$$

$$-2ik_P\frac{dA^P}{dz} + \frac{d^2A^P}{dr^2} - 2k_P^2\Delta\left(\frac{r}{R}\right)^2 A^P = -2k_P^2\frac{n_2}{n_0}(|A^P|^2 + (2-if)|A^S|^2)A^P,$$

where A^S and A^P are the field envelopes of the Stokes and pump waves, respectively, $k_{S|P} = 2\pi n_{co}/\lambda_{S|P}$ is the wavenumber, and $f = 0.2$ is a Raman and Kerr constants ratio.

Let us introduce the following dimensionless and normalized variables:

$$\Delta_\beta = \frac{\sqrt{2\Delta}}{R}, \zeta = z\Delta_\beta, A^{S,P} = \Psi^{S,P}\sqrt{P^{S,P}}.$$

In this notation, the normalized field envelope can be decomposed into a sum of spatial Laguerre modes as:

$$\Psi^S(\zeta,\vec{r}) = \sum_{m,p=0}^{\infty} A_{p,m}(\zeta) U_{p,m}^S(\vec{r}),$$

$$\Psi^P(\zeta,\vec{r}) = \sum_{m,p=0}^{\infty} B_{p,m}(\zeta) U_{p,m}^P(\vec{r}),$$

where

$$U_{p,m}^{S,P}(\vec{r}) = N_{p,m} \frac{r^{|m|}}{\rho_{S,P}^{|m|+1}} L_p^{|m|}\left(\frac{r^2}{\rho_{S,P}^2}\right) exp\left(-\frac{r^2}{2\rho_{S,P}^2}\right) exp(im\phi),$$

$$N_{p,m} = \sqrt{\frac{p!}{\pi(p+|m|)!}}, \rho_{S,P} = \frac{1}{\sqrt{k_{S,P}\Delta_\beta}}.$$

Here $A_{p,m}$ and $B_{p,m}$ are mode amplitudes of the Stokes and pump waves, respectively, p and m are radial and azimuthal mode orders, and $L_p^{|m|}$ are Laguerre polynomials.

In this case, using the orthonormality of the spatial distribution of the modes and neglecting rapidly oscillating term, we can derive a system of propagation equations for mode amplitudes of the pump wave and the Stokes component in GRIN MMF:

$$\frac{\partial A_{p,m}}{\partial z} = \sum_{m1,p1} C_{m,p}^{m1,p1} A_{m1,p1} - \frac{\alpha_S}{2} A_{p,m} -$$

$$-i\frac{k_S n_2}{\Delta_\beta n_{co}} \sum_{m1,m2,m3} \sum_{p1,p2,p3} \left(q_{p1,p2,p3,p}^{m1,m2,m3,m} P_S A_{p1,m1}^* A_{p2,m2}\right.$$

$$\left. + g_{p1,p2,p3,p}^{m1,m2,m3,m} P_P (2+if) B_{p1,m1}^* B_{p2,m2}\right) A_{p3,m3}$$

$$\frac{\partial B_{p,m}}{\partial z} = \sum_{m1,p1} C_{m,p}^{m1,p1} B_{m1,p1} - \frac{\alpha_P}{2} B_{p,m} -$$

$$-i\frac{k_P n_2}{\Delta_\beta n_{co}} \sum_{m1,m2,m3} \sum_{p1,p2,p3} \left(h_{p1,p2,p3,p}^{m1,m2,m3,m} P_P B_{p1,m1}^* B_{p2,m2}\right.$$

$$\left. + l_{p1,p2,p3,p}^{m1,m2,m3,m} P_S (2-if) A_{p1,m1}^* A_{p2,m2}\right) B_{p3,m3}.$$

Fibre losses for the Stokes ($\alpha_S = 2.64$ dB/km) and pump ($\alpha_P = 2.72$ dB/km) waves were also included here. The coefficients $q_{p1,p2,p3,p}^{m1,m2,m3,m}$, $g_{p1,p2,p3,p}^{m1,m2,m3,m}$, $h_{p1,p2,p3,p}^{m1,m2,m3,m}$, and $l_{p1,p2,p3,p}^{m1,m2,m3,m}$ are obtained by calculating the overlap integrals of the spatial modes of the pump waves and the Stokes component.

This coupled mode model allows one to take into account nonlinear effects of self-phase modulation, cross-phase modulation, Raman effects. In addition to the nonlinear effects and fibre loss, this system of equations also takes into account the random linear coupling between all spatial modes due to various fibre imperfections, bends and stresses, see [S2] for more details. We consider the random coupling between all modes and coefficients $C_{m,p}^{m1,p1}$ are normally distributed random numbers with zero mean and standard deviation σ. In the simulations we consider 496 modes with the mode number n = 2p + |m| $\leq$ 30.

For the initial conditions for the Stokes wave, we used the decomposition of a Gaussian beam with a radius of 12 μm into spatial modes, we also initially set a random phase for each mode. For the pump wave, all modes have equal intensities and random phases. Calculations were performed for different realizations of random phases, and then the results were averaged.

For a numerical solution, the system of equations was written in matrix form:

$$\frac{\partial A}{\partial z} = M_A A,$$

$$\frac{\partial B}{\partial z} = M_B B,$$

where A and B are vectors containing the amplitudes of all Stokes and pump modes, respectively, and the matrices M_A and M_B correspond to the right-hand side of the coupled mode model. The solution at each next step of integration can be found using the following numerical scheme:

$$A^{n+1} = e^{hM_A^n} A^n,$$

$$B^{n+1} = e^{hM_B^n} B^n,$$

where A^n and B^n are the solution at the nth step; and h is the integration step. The matrix exponent can be calculated using the Padé approximation.

In our simulation we consider the multi-pass problem. For the first pass for the Stokes wave, the described Gaussian beam was used at the input. To match the physical experiment and for taking into account the FBG, we added 4% filter of the fundamental mode at the fibre output. Hence, after each pass, the amplitude of the fundamental mode of the Stokes wave was multiplied by $\sqrt{0.04}$ (corresponds to the filter after propagation in both directions), and the other modes were multiplied by $\sqrt{0.004}$; the resulting beam was used as the input data for the next pass. For the pump wave, all modes were initialized with equal intensities and random phases at the input of each pass.

As a first step, we have checked that summing up all the modes with random phases and equal powers (equipartition case) gives nearly rectangular and nearly parabolic profiles in SIFs and GIFs, respectively. We have also checked that random mode coupling (with the coefficient of linear coupling strength $s = 2\cdot10^{-7}$ corresponding to experiment) leads to the conversion of rectangular to parabolic profile after propagation of ~10 m of GIF (see Fig.S4), just like in experiment after pump combiner.

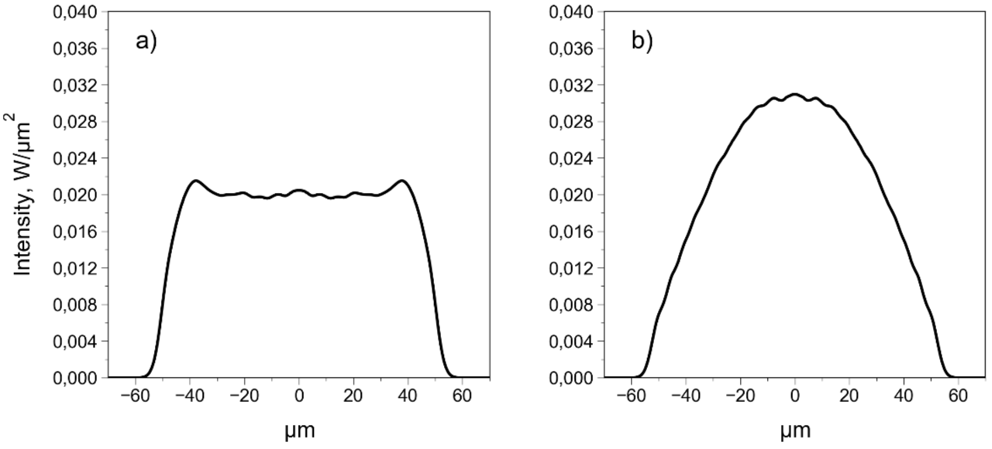

Fig.S4. Spatial distribution obtained by decomposing an equidistributed rectangular beam of a step index fibre with a diameter of 80 μm into 861 Laguerre modes of the considered GIF (a), and output distribution of the beam after 10 m propagation along the GIF with random linear mode coupling (b).

The beam profile remains parabolic when propagating in 1 km GIF below Raman threshold. At the Raman threshold (P_{in} ~110 W), the Stokes beam appears at the output, without significant changes of the output pump beam. In Fig.S5 we compare different propagation regimes near Raman threshold. We consider the propagation taking into account only the Raman effect and without fundamental mode filter (violet curves); propagation with Raman and Kerr effects and random linear coupling with (green curves) and without filter (bleu curves); and propagation taking in account the Raman effect and random linear coupling without FM filter (red curves). In the case of considering only the Raman effect, after convergence, almost all the energy of the Stokes component is contained in the fundamental mode (more than 99%), and the output pump and Stokes beams power is 58.2 W and 0.68 W, respectively. When random linear coupling is included in the model, the output Stokes power reaches 1.44 W, while the content of FM is halved (51%). The output pump power for this case is 57.5 W. Taking into account Kerr effect, together with random linear mode coupling and Raman effects, leads to a decrease in the output Stokes power to 0.86 W and a slight increase in the pump power to 58 W. In this case, the energy content in the fundamental mode for the Stokes beam decreases to 43%.

When a fundamental mode filter is added, the energy contained in high-order modes is practically not reflected after each pass, and the FM content increases to 75%. The output pump and Stokes beams power for this case are 58.2 W and 0.64 W, respectively. Fig S5a and Fig S5b show the output distributions of intensities of the pump wave and the Stokes component for considered regimes, respectively. In Fig S5a, the considered cases are also compared with the output distribution of the pump beam, which was affected only by losses during propagation (black curve). For this case, the output beam power is 58.8 W. Due to the fact that for all cases the Stokes component is amplified insignificantly in comparison with the pump power, all spatial profiles in Fig S5a are similar to each other. Fig S5c shows the difference between the output and input pump intensity distributions that manifests a rather broad distribution of the depleted pump in all the treated cases, with a slightly different structure of the depleted transverse modes in the pump beam.

At the same time, the generated Stokes beam has nearly the same shape (near-Gaussian) with predominant content (>40%) of the fundamental mode in all the cases. It should be noted that for Fig S5b and Fig S5c spatial distributions are normalized to 1 W.

The situation changes well above the threshold, when pump depletion becomes important, resulting in quite different Stokes beam shapes in the treated cases (see Fig.S6). When modeling a pure pump-to-Stokes Raman conversion (Raman beam cleanup effect) in a non-filtering cavity, one obtains spatial beam profiles (violet curves) exhibiting a modulation, which arises from the mode structure of both Stokes and depleted pump beams: such spatial modulation is absent in the experiment, at that the content of FM is ~10% only. In this case, after convergence, the Stokes beam is amplified to about 37 W, while the output pump power is 49 W: these values are also far from the experimental data.

Adding random mode coupling for both beams (red curves) washes out the modulation so that the depleted pump beam becomes closer to the experiment. However, one also obtains a smooth multimode Stokes beam (with the FM content reduced to ~7%) which is much broader than in the experiment. Adding linear mode coupling also increases the output Stokes beam power to 46 W while the pump wave has the power of 39 W at the fibre output.

The inclusion of the Kerr effect (blue curves) permits to significantly compress the width of the Stokes beam with the FM content increased to 31%. The Kerr-cleaning effect, in addition to increasing the part of the energy contained in the fundamental mode, also leads to a decrease in the output Stokes power to 38 W. The output pump power for this case is 44 W. At last, adding spatial filtering of the fundamental mode (with 10 dB suppression of high-order modes) permits to significantly improve the Stokes beam quality (with the FM content increased to ~70%), so that it becomes close to the experiment both in shapes and power values. In this case, almost all the energy contained in HOMs is dissipated after each pass, and the output Stokes power is 29 W, while the output power of the pump beam is increased to 54 W.

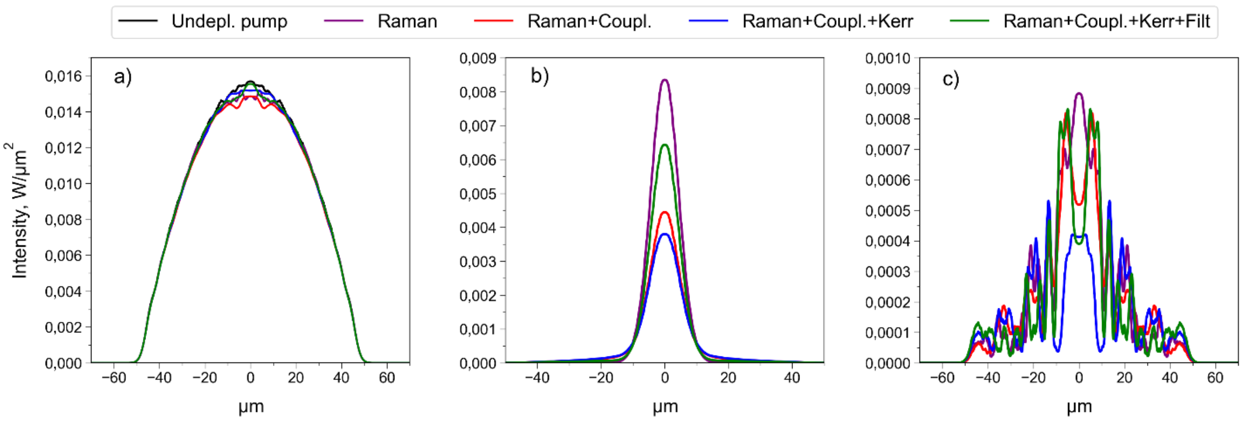

Fig.S5. Theoretical comparison of spatial profiles for pump (a) and Stokes (b) beams, and dip in the pump (c) for different propagation regimes near the Raman threshold (normalized to 1 W Stokes power).

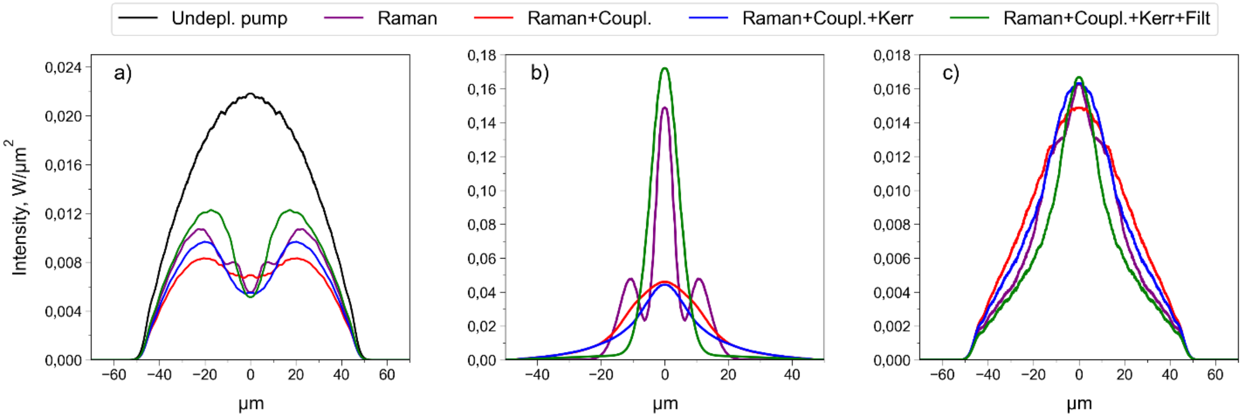

Fig.S6. Theoretical comparison of spatial profiles for pump (a) and Stokes (b) beams and dip in the pump (c) for different propagation regimes well above the Raman threshold.

References

[S1] A.G. Kuznetsov, S.I. Kablukov, E.V. Podivilov, S.A. Babin, "Pump depletion in Raman lasing in a graded index multimode fibre," Quant. Electronics **50** (12), 1091–1095 (2020).

[S2] O.S. Sidelnikov, E.V. Podivilov, M.P. Fedoruk, S. Wabnitz, "Random mode coupling assists Kerr beam self-cleaning in a graded-index multimode optical fiber," Opt. Fiber Technology **53,** 101994 (2019).
